# A reminder of the place of morphology and the H‐score in the diagnosis of hemophagocytic lymphohistiocytosis (HLH)

**DOI:** 10.1002/ccr3.1391

**Published:** 2018-02-06

**Authors:** Julien Favresse, Benjamin Lardinois, Bernard Chatelain, François Mullier, Hugues Jacqmin

**Affiliations:** ^1^ Hematology Laboratory CHU UCL Namur Université catholique de Louvain Yvoir Belgium

**Keywords:** hemophagocytic syndrome, hemophagocytosis, HLH, H‐score

## Abstract

This case report reminds the reader of the place of hemophagocytosis and the H‐Score in the diagnosis of secondary hemophagocytic lymphohistiocytosis.

This case report aimed to remind readers of the place of hemophagocytic features in the diagnosis of secondary hemophagocytic lymphohistiocytosis or hemophagocytic syndrome. Integration of hemophagocytosis along with other criteria (e.g., immunosuppressive state, extent of cytopenia, or ferritin) is mandatory as hemophagocytic macrophages may also be present in the absence of proven hemophagocytic syndrome (e.g., sepsis, blood transfusion). For that purpose, we opted to use the H‐Score which has been validated in adults to identify the probability of hemophagocytic syndrome. We also discuss the limitation of the well‐known HLH‐2004 score in comparison with the H‐Score which appeared be more sensitive and easier to calculate in routine practice.

A 70‐year‐old man with a Wegener's granulomatosis (anti‐PR3 183 UI/mL, normal range (NR) <5.1 UI/mL) treated with corticosteroids and cyclophosphamide was admitted to our hospital to investigate the development of acute renal failure, along with fever (peak 38.2°C) and inflammation (CRP 34.5 mg/L, NR <5 mg/L). The kidney biopsy showed no focal necrotizing glomerulonephritis, and a hypothesis of hemophagocytic lymphohistiocytosis (HLH) was supported by the presence of cytopenias (hemoglobin 99 g/L, NR 133–176 g/L and platelets 58 × 10^9^/L, NR 150–400 × 10^9^/L), elevated serum concentrations of ferritin (17,400 μg/L, NR 17.7–464 μg/L), and to a lesser extent elevated triglyceride (1.91 mmol/L, NR <1.69 mmol/L). The bone marrow aspirate showed an increased number of hemophagocytic macrophages characterized by the phagocytosis of nucleated cells 1 (Figs 1 and 2).

**Figure 1:**
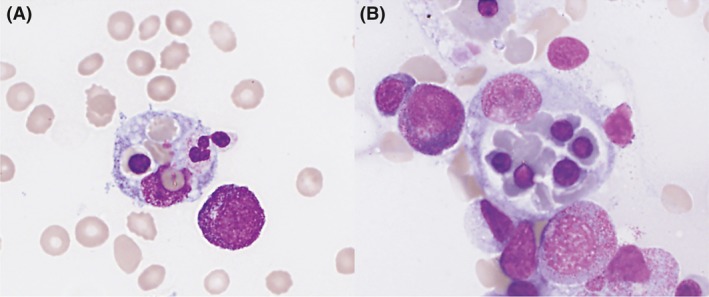
Bone marrow aspirate showing two macrophages that have ingested (A) three red blood cell, a polychromatophilic erythroblast and a neutrophile and (B) five polychromatophilic erythroblasts and red blood cells. May‐Grünwald Giemsa stain ×40.

**Figure 2:**
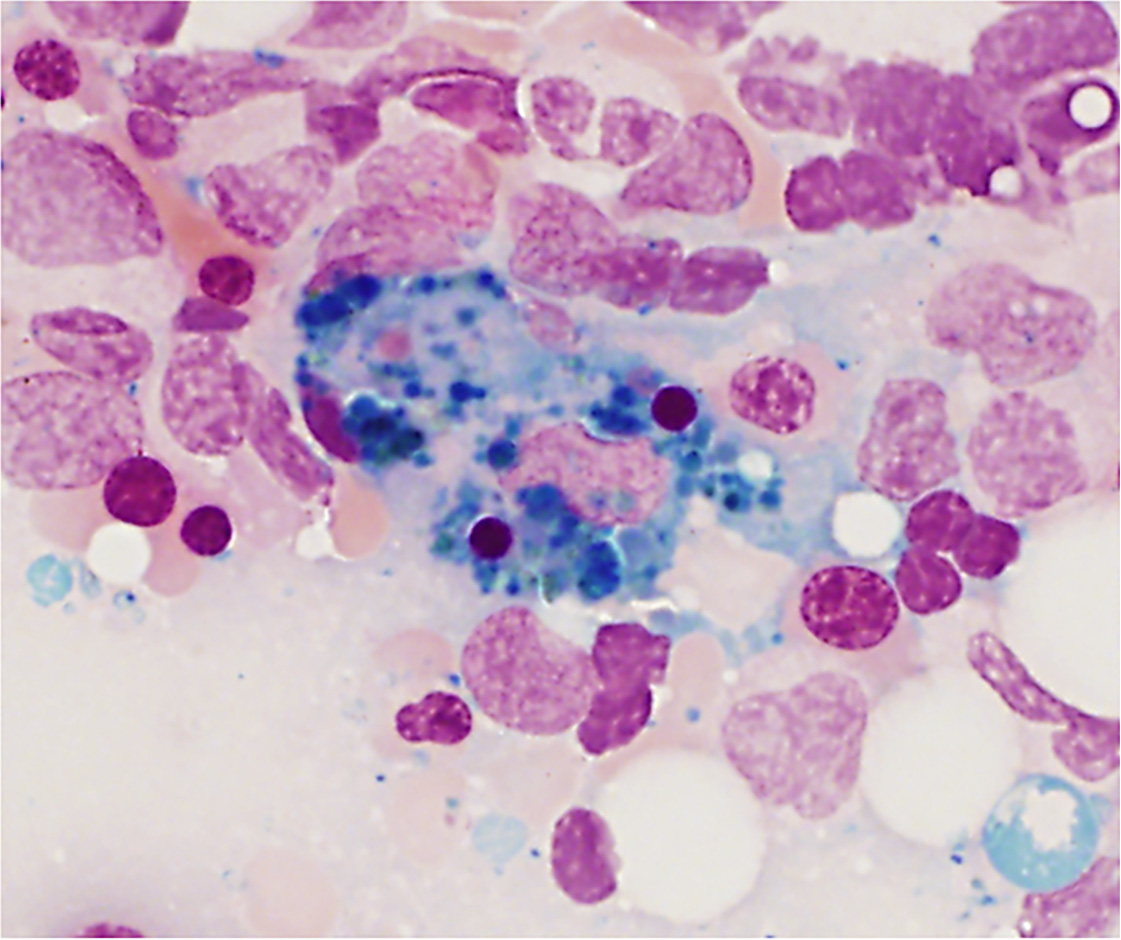
Bone marrow aspirate showing an iron‐laden macrophage that has ingested two polychromatophilic erythroblasts. Perls's stain ×40.

HLH, also called hemophagocytic syndrome, is a rare clinical syndrome caused by a highly stimulated but ineffective immune response. HLH may be the consequence of an inherited (underlying genetic disorder) or acquired (associated with malignancy, autoimmune disease, or infection) inability of the immune system to eliminate a trigger 1, 2. Macrophage activation syndrome (MAS) is also a form of HLH but is restricted to underlying rheumatic diseases mostly in children 2, 3.

It is important to keep in mind that hemophagocytic macrophages may also be present in the absence of proven hemophagocytic syndrome (e.g., sepsis, blood transfusion) 4. Therefore, multiple criteria should be considered, and various scoring systems exist to identify the probability of hemophagocytic syndrome 4, 5.

We opted for the H‐Score because it has been validated in adults, and the measurements required are more easily available in routine practice (immunosuppressive state, maximal temperature, hepatomegaly, splenomegaly, extent of cytopenia, ferritin, triglyceride, fibrinogen, ASAT, and the presence of hemophagocytosis features on bone marrow aspirate) compared to the HLH‐2004 scoring system which requires assessment of natural killer cell activity or NK cell degranulation and the measurement of the *α*‐chain of the soluble interleukin‐2 receptor 4. Moreover, the H‐Score appeared be more sensitive than the HLH‐2004 score for rheumatic diseases but with similar specificity 6. Besides the elevation of ferritin and triglyceride, ASAT (161 U/L, NR 17–59 U/L) and LDH were elevated (1921 U/L, NR 313–618 U/L). The leukocyte count (4.74 × 10^9^/L NR 3.70–9.50 × 10^9^/L) and fibrinogen level (2.78 g/L, NR 1.80–4.00 g/L) were normal. The H‐Score was calculated online and gave a probability of 96% (http://saintantoine.aphp.fr/score/).

In this case, the underlying autoimmune disease in addition to the patient's immunosuppressed status is the most likely triggers of this secondary HLH.

## Authorship

FJ, LB, CB, MF, and JH: analyzed and interpreted the biological, hematological, and clinical data, read and approved the final manuscript. FJ and LB: contributed manuscript writing. HJ: supervised manuscript writing and submission.

## Conflict of Interest

None declared.
